# Correlation of sperm penetration assay score with polyspermy rate in in-vitro fertilization

**DOI:** 10.1186/1743-1050-2-3

**Published:** 2005-02-09

**Authors:** Vincent W Aoki, C Matthew Peterson, Kirtly Parker-Jones, Harry H Hatasaka, Mark Gibson, Ivan Huang, Douglas T Carrell

**Affiliations:** 1 Andrology and IVF Laboratories, University of Utah School of Medicine, Salt Lake City, UT, 84108, USA; 2 Department of Surgery, University of Utah School of Medicine, Salt Lake City, UT, 84132, USA; 3 Department of Physiology, University of Utah School of Medicine, Salt Lake City, UT, 84132, USA; 4 Department of Obstetrics and Gynecology, University of Utah School of Medicine, Salt Lake City, UT, 84132, USA

## Abstract

**Background:**

The sperm penetration assay (SPA) is used to predict the fertilizing capacity of sperm. Thus, some programs rely on SPA scores to formulate insemination plans in conjunction with in-vitro fertilization (IVF) cycles. The purpose of this study was to evaluate if a relationship exists between SPA scores and polyspermy rates during conventional IVF cycles.

**Methods:**

A total of 1350 consecutive IVF patients using conventional IVF insemination were evaluated in the study. Oocytes were inseminated three hours post-retrieval by the addition of 150,000 to 300,000 progressively motile sperm. Approximately 18 hours after insemination, the oocytes were evaluated for fertilization by the visualization of pronuclei. The presence of three or more pronuclei was indicative of polyspermy. Polyspermy rates, fertilization success, embryo quality, and pregnancy rates were analyzed retrospectively to evaluate their relationship with SPA score, count, motility, number of progressively motile sperm inseminated, oocyte pre-insemination incubation time, patient age, and diagnosis.

**Results:**

A significant positive relationship was observed between SPA score and polyspermy rate (r_s _= 0.10, p < 0.05). Patients with a normal SPA score had significantly higher polyspermy rates than those with abnormal SPA scores (6.3% ± 1.5% vs. 2.0% ± 0.7%, p < 0.05). Fertilization percentage was significantly lower in the group with severely abnormal SPA scores versus all other SPA groups (57.5% ± 2.1% vs. 70.2% ± 1.3%, p < 0.005). Although embryo quality was not affected, both clinical pregnancy and implantation rates improved slightly as SPA score increased. In addition, there was a decrease in the rate of spontaneous abortion as SPA score increased.

**Conclusions:**

These data indicate SPA score is positively correlated with polyspermy rates and IVF fertilization percentage. Additionally, there is a slight increase in clinical pregnancy rates, and embryo implantation rates with increased SPA. Furthermore, there is a slight decrease in spontaneous abortions rates related to increased SPA.

## Findings

The sperm penetration assay (SPA) is used to evaluate the fertilizing capacity of human spermatozoa [1]. Some in-vitro fertilization (IVF) programs rely on the SPA to formulate their insemination plans in conjunction with IVF cycles [1,2]. Couples with normal SPA scores normally undergo conventional IVF insemination in contrast with intracytoplasmic sperm injection (ICSI), which is used for those with a diminished SPA.

Over the past decades success rates have steadily improved for human IVF embryo transfer programs. Recent advances in ovulation induction, micromanipulation, culturing conditions, and media formulations have fostered a technological revolution for IVF leading into the 21'st century (Pool, 2002). However, many common problems still remain.

One common problem associated with conventional IVF insemination is polyspermic fertilization when more than one sperm successfully penetrates and fertilizes the oocyte. These pre-embryos are considered abnormal and typically discarded. Thus, increased rates of polyspermy ultimately result in a reduced number of embryos.

Studies have been conducted that evaluate the factors associated with human IVF polyspermy [3-5]. However, most of the reports are based on data over a decade old. Recent improvements in IVF techniques may alter these relationships. Furthermore, no study has evaluated the relationship between sperm SPA score and incidence of polyspermy in human IVF.

The purpose of this study was to evaluate if a relationship exists between SPA scores and polyspermy rates during IVF cycles utilizing conventional insemination. We also evaluated the relationship between SPA score and fertilization rate, embryo quality, and pregnancy rate.

After Institutional Review Board approval, we conducted a retrospective study of 1350 consecutive IVF patients using conventional IVF insemination. Intracytoplasmic sperm injection (ICSI) patients were excluded from the study. The fertilizing capacity of each patient was assessed with the SPA. The SPA was performed on patients undergoing IVF using techniques previously described [6]. The SPA was performed on patients less than sixty days prior to the IVF cycle. The SPA score reflects the percentage of eggs successfully penetrated by the patient's sperm. SPA patients were stratified into 4 groups according to SPA score (Group 1: severely abnormal SPA (< 10% penetration), n = 182; Group 2: abnormal SPA (10–19% penetration), n = 368; Group 3: normal SPA (20 – 29% penetration), n = 404; Group 4: high-normal SPA (> 30% penetration), n = 396).

During the IVF cycle ovarian stimulation was performed using standard techniques of gonadotrophin-releasing hormone (GnRH) agonist down-regulation combined with controlled ovarian stimulation using a combination of recombinant follicle stimulating hormone (rFSH) and urinary-derived gonadotropin stimulation. Ovarian follicles were aspirated using a trans-vaginal ultrasound-guided needle.

Oocytes were inseminated three-hours post-retrieval by the addition of 150,000 to 300,000 progressively motile sperm. Approximately 18-hours post-insemination, the oocytes were evaluated for fertilization by visualization of pronuclei. The presence of three or more pronuclei was indicative of polyspermy in which case the fertilized oocyte was discarded. Embryos were cultured in HTF medium and transferred 72-hours post-retrieval. Embryo quality was assessed using a previously reported embryo scoring system that took into account the number of cells present and the level of cellular fragmentation [7].

Polyspermy rates, fertilization success, embryo quality, and pregnancy rates were analyzed retrospectively with respect to their relationship with SPA score. The correlation between SPA score and polyspermy was conducted using Spearman's correlation coefficient. Additionally, polyspermy rates, fertilization percentage, and embryo quality for the different SPA groups were analyzed for statistical difference using Kruskal-Wallis analysis. Lastly, pregnancy rates in the different groups were evaluated with a Chi-square analysis.

A significant positive relationship was observed between SPA score and polyspermy rates in 1350 IVF patients undergoing conventional IVF insemination (r_s _= 0.10, p < 0.05). Patients with normal SPA scores (Group 3) had significantly elevated polyspermy rates over those with severely abnormal SPA scores (6.3 ± 1.5 – 113/1,791 vs. 2.0 ± 0.7 – 20/1,020, p < 0.05, Table 1, please see file "Table1-Aoki et al.doc", Additional file 1). Conventional IVF fertilization rate was significantly lower in patients with severely abnormal SPA (57.5 ± 2.1 – 1,135/1,974) vs. all other patients (70.2 ± 1.3 – 8,519/12,135; p < 0.005, Table 1, please see file "Table1-Aoki et al.doc", Additional file 1).

Embryo quality showed no relationship to the SPA (Table 1, please see file "Table1-Aoki et al.doc" Additional file 1). Clinical pregnancy and implantation rates were increased in Group 4 (50.8% – 202/396 and 27.4% – 343/1255, respectively) versus Group 1 (31.6% – 58/182 and 15.7% – 96/612, respectively, p < 0.05) but no significant relationship between SPA and IVF outcome was observed throughout the entire study population (Table 1, please see file "Table1-Aoki et al.doc" Additional file 1). Similarly, there was a decrease in the rate of spontaneous abortion in Group 4 (14.7% – 30/202) versus Group 1 (31.3% – 18/58), although no significant relationship was observed within the entire population (Table 1, please see file "Table1-Aoki et al.doc" Additional file 1).

No relationships were observed between IVF polyspermy rates and maternal age, maternal diagnosis, amount of progressively motile sperm added, sperm morphology, ovarian stimulation protocol, or post-oocyte retrieval pre-insemination incubation time. Additionally, confounder analysis indicated the SPA groups were similar with respect to paternal age, maternal age and IVF diagnosis. Therefore, no standardization of the data with respect to these variables was required.

This is the first report to correlate SPA score with IVF polyspermy rates. Significantly elevated IVF polyspermy rates were observed in patients with normal SPA scores. Previous reports have established a relationship between IVF polyspermy and oocyte age/maturation, state of the zona pellucida, number of progressively motile sperm used for insemination, oviductal cytokine expression, and in-vitro culture conditions such as pH, temperature, and media supplementation [5].

Unlike other reports, we did not find a relationship between the number of progressively motile sperm used for insemination and IVF polyspermy rates. This discrepancy is most likely due to the narrow range of sperm number we used for insemination (150,000 to 300,000) compared with the studies of van der Ven *et al. *who used 500,000 to 1.5 *10^6 ^sperm [4]. Our results are consistent with Ho *et al. *who found no relationship between these variables [3]. However, the data still suggest minimizing the number of sperm added for IVF insemination in patients with increased SPA scores.

Poly-pronuclear formation in in-vitro fertilized eggs usually arises from polyspermic fertilization but may also be a product of second polar body retention [8]. Thus, one potential pitfall of associating poly-pronuclear fertilized oocytes with polyspermy is the uncertainty related to second polar body retention. However, a recent report showed only a small percentage of fertilized oocytes with three-pronuclei (2.5%) arise from second polar body retention. The findings of this study validate our study design of categorizing poly-pronuclear fertilized oocytes as polyspermic. Moreover, we would expect the contribution of second polar body retention to be consistent throughout the SPA groups negating any confounding effects.

Consistent with other reports, the SPA was correlated with fertilization percentage [1,6]. Furthermore, clinical pregnancy and implantation rates did show improvement in patients with a high-normal SPA versus those with a severely abnormal SPA. In addition, spontaneous abortion rates were significantly different between these two groups. This data strongly suggests ICSI may be an attractive alternative for patients with severely affected penetration ability.

Done correctly, the SPA provides a reliable assessment of the fertilizing ability of human spermatozoa with very low false negative rates (< 0.03%) and serves a valuable tool for clinicians to treat infertility patients with the appropriate modality [9]. The SPA is particularly useful in light of recent concerns about ICSI and imprinting diseases [10-12]. These concerns have led to the recommendation that IVF clinicians should be careful to employ the technique only when necessary and the SPA provides a prognostic tool to appropriately make that decision. Based on these findings, it may be valuable for individual IVF programs to re-evaluate the clinical utility of SPA testing. The SPA may prove valuable for clinicians to avoid increased amounts of polyspermy and unnecessary wasting of oocytes. Meanwhile, the SPA does not appear to be a reliable indicator of IVF embryo quality or pregnancy rates.

## Authors' Contributions

VWA designed the study, carried out statistical evaluation, and was the primary author of the manuscript. CMP, KPJ, HHH, MG, and IH were involved in management of the IVF cases and collection of the clinical data. DTC was responsible for the implementation of the study, administrative requirements including IRB approval, statistical evaluation of the data, and preparation of the manuscript.

## Supplementary Material

Additional File 1 Table 1. Relationship between SPA and IVF outcome measures Click here for file
