# Supplementary material for: Correlation of sperm penetration assay score with polyspermy rate in in-vitro fertilization
Source: J Exp Clin Assist Reprod. 2005 Feb 9;2:3. doi: 10.1186/1743-1050-2-3 (PMC549527; doi:10.1186/1743-1050-2-3)
Supplement: Additional File 1 — Table 1. Relationship between SPA and IVF outcome measures [file 1743-1050-2-3-S1.doc]

# Table 1. Relationship between SPA and IVF outcome measures.

|  | Group 1  (Severely Abnormal SPA) | Group 2  (Abnormal SPA) | Group 3  (Normal SPA) | Group 4  (High-Normal SPA) | Significance |
| --- | --- | --- | --- | --- | --- |
| N | 182 | 368 | 404 | 396 |  |
| Polyspermy Rate (%) | 2.0 ± 0.5^*^ | 4.3 ± 0.7^*^ | 6.3 ± 0.8 | 5.7 ± 0.9 | p < 0.05^*^ |
| Fertilization Rate (%) | 57.5 ± 2.2^+^ | 67.7 ± 1.3 | 72.3 ± 1.2 | 70.3 ± 1.5 | p < 0.005^+^ |
| Embryo Quality | 4.3 ± 0.3 | 4.2 ± 0.1 | 4.3 ± 0.1 | 4.1 ± 0.1 | NS |
| Pregnancy Rate (%) | 31.6^#^ | 42.1 | 45.2 | 50.8 | p < 0.05^#^ |
| Implantation Rate (%) | 15.7^#^ | 12.2 | 11.6 | 27.4 | p < 0.05^#^ |
| Spontaneous Abortion Rate (%) | 31.3^#^ | 26.9 | 25.8 | 14.7 | p < 0.05^#^ |
| Maternal Age | 34.7 ± 0.4 | 33.8 ± 0.3 | 34.3 ± 0.2 | 34.1 ± 0.3 | NS |

* Significantly reduced versus groups 3 and 4

+ Significantly reduced versus all other groups

# Significantly reduced or increased versus group 4
